# Behavior of Ants Escaping from a Single-Exit Room

**DOI:** 10.1371/journal.pone.0131784

**Published:** 2015-06-30

**Authors:** Shujie Wang, Wei Lv, Weiguo Song

**Affiliations:** 1 State Key Laboratory of Fire Science, University of Science and Technology of China, Hefei, Anhui, China; 2 Center of Crisis and Hazard Research, Wuhan University of Technology, Wuhan, Hubei, China; National Scientific and Technical Research Council (CONICET)., ARGENTINA

## Abstract

To study the rules of ant behavior and group-formation phenomena, we examined the behaviors of *Camponotus japonicus*, a species of large ant, in a range of situations. For these experiments, ants were placed inside a rectangular chamber with a single exit that also contained a filter paper soaked in citronella oil, a powerful repellent. The ants formed several groups as they moved toward the exit to escape. We measured the time intervals between individual escapes in six versions of the experiment, each containing an exit of a different width, to quantify the movement of the groups. As the ants exited the chamber, the time intervals between individual escapes changed and the frequency distribution of the time intervals exhibited exponential decay. We also investigated the relationship between the number of ants in a group and the group flow rate.

## Introduction

### 1 Ants under normal conditions

As social insects, ants are self-propelled agents that react to their surroundings. They are also relatively easy to control. For these reasons, ants are favored by researchers studying the behaviors of social insects. Many studies have focused on ant social organization [1,2] and traffic organization [3–8] under normal conditions and have provided valuable insight.

For example, with regard to social organization, an ant migration algorithm has been developed that can be used to study the collective motion of ants [1]. The development of an organized society in an ant colony has also been investigated, such as in Mersch et al.’s study [2], which investigated how the division of labor among workers is formed. The movement of ants has been investigated from the perspective of traffic organization. Burd and colleagues reported that an increasing encounter rate decreased ant speed but that head-on encounters could result in the exchange of information between workers; this form of traffic organization was found to be superior to segregated outbound and return lanes in some situations [3]. A model of bi-directional ant traffic in pre-existing ant trails has been proposed, suggesting that the flow rate becomes approximately constant over some density intervals as a consequence of the interactions between ants moving in opposite directions [5]. Examinations of ant movement patterns have also shown that ants do not experience jamming in unidirectional, single-lane trails [8].

### 2 Ants under stress conditions

To consider the influence of stress conditions, Altshuler et al. [9] conducted experiments of leaf-cutting ants under the effect of a repellent. These authors found that ants displayed a “symmetry breaking” phenomenon: when under stress, they escaped from a chamber with two symmetrically located exits by using one exit more often than the other. Because it is relatively simple to measure the behavior of non-human animal species, ants have been proposed as a model for humans [10]. However, the egress behaviors of ants have been shown to differ from those of many other animals and humans [11–13]. Our goal in this work is to further confirm this difference.

Soria et al. [11] investigated the dynamic phenomenon “Faster is Slower” in the carpenter ant *Camponotus mus*, which refers to the fact that evacuation time decreases with increasing speed up to a threshold point; beyond this threshold, increased speed leads to increasing evacuation time. However, the behavior of ants differs from that expected based on a model simulating human behavior [14]. In simulations of pedestrian behavior, each pedestrian is expected to attempt to escape as quickly as possible when an evacuation begins, yet this type of behavior produces a jam, which reduces the efficiency of the evacuation. In contrast, ants do not follow a direct path toward an exit, nor do their maneuvers produce jamming near an exit. In an investigation of the behavior of ants escaping from temperature stress, Boari et al. [12] found no evidence of “selfish evacuation behavior”, providing experimental support for the “Faster is Faster” effect, as opposed to the “Faster is Slower” effect. Parisi et al. [13] later showed that ants did not form high densities near an exit; thus, the nature of the “Faster is Slower” effect observed in ants [11] was different from that proposed by the Social Force model [14].

In general, experiments of ant behavior in stress situations are attractive because they add to our understanding of animal welfare [15,16], a topic that has received increasing attention in recent years. Nonetheless, the evacuation behavior of ants is worth studying for its own unique characteristics. For example, ants evacuate efficiently and do not produce jamming or clogging [11–13], which differs from human behavior. In the present study, we investigated the escape behavior of *Camponotus japonicus* from a chamber with one exit of varying width. The phenomenon of group formation is very general and is widely studied for clogging in discrete systems [14]. Furthermore, comparisons of this type of data have not been made with respect to humans and ants in previous work. In this paper, the general characteristics of ants are studied and compared to those of humans to determine whether they are similar or not.

## Materials and Methods

### Ethics Statement

The study was approved by the Animal Research Committee of the University of Science & Technology of China (USTC) (Permit Number: USTCACUC1309018). All efforts were made to minimize suffering.

### Insects

We used multiple colonies of *C*. *japonicus*, which were captured in the wild in Shaanxi Province (33°N, 107°E) (China) and transported to the laboratory. The ants were placed in nests constructed of plaster material that is suitable for ants, and the tops of the nests were covered by glass. The nests were maintained in the laboratory under natural light/dark cycles and nearly constant temperature (24±4°C). The ants were fed fruit and honey when they were not participating in consecutive experiments. To estimate the size of the population and calibrate the measurement chambers, the head width (i.e., the largest lateral dimension of the individual’s head) and body length of the ants were measured using a ruler to an accuracy of 0.1 cm.

### Experimental chamber

**Fig 1:**
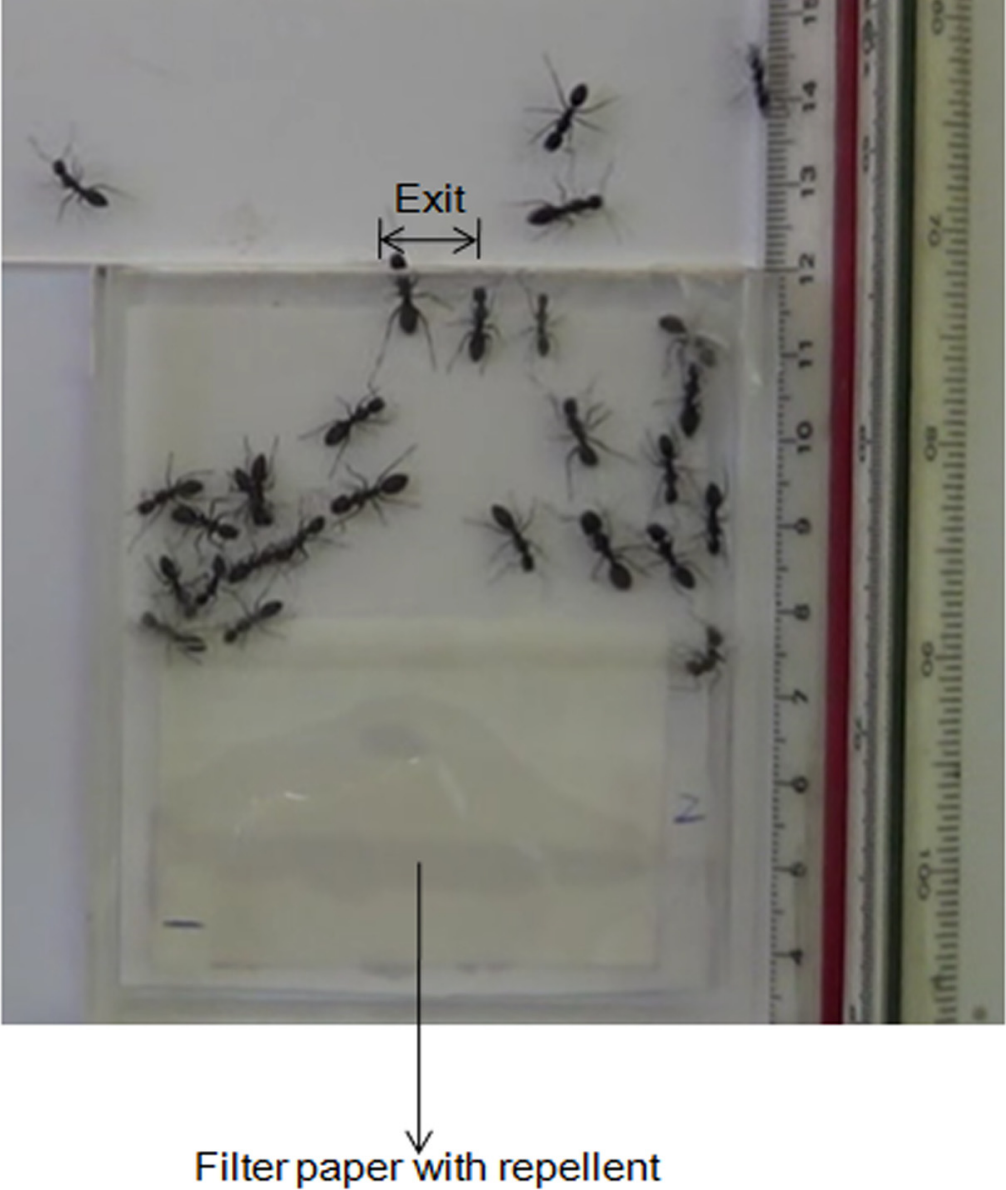
Video recording of ants evacuating from a single-exit room. The exit width is 2*w* = 1.0 cm, and a 3.3% concentration of citronella was used.

We built a chamber consisting of a rectangular PMMA (polymethyl methacrylate) box (7.0 cm wide × 8.0 cm long), as shown in Fig 1. The entire chamber was covered by two PMMA lids, leaving a height of 0.6 cm to prevent the ants from climbing on top of each other. On one side of the chamber was a single exit of varied width. A repellent substance (citronella oil) was used to induce stress in the ants and drive them toward the exit; a filter paper that had been soaked in the repellent substance was placed in the chamber near the side opposite to the exit.

### Experimental protocol

The experiment was carried out in two parts; first, as an evacuation experiment in which the repellent substance was included; second, as a control experiment in which pure water was used rather than the repellent. We varied the width of the exit (0.5, 1.0, 1.5, 2.0, 2.5, or 3.0 cm) in both experiments. In experiment 1, we placed 30±2 ants of similar size from the same nest into the rectangular chamber; the exit had been blocked. After the ants were introduced, the chamber was covered by the PMMA lids, and we added 700 μL of 3.3% citronella oil and then opened the exit so that the ants could escape. When the citronella oil was present, all of the ants egressed. Each ant was exposed to the experimental treatment only once, and the experimental sessions were recorded using a digital video camera (digital sampling rate = 25 frames per sec). Each session began from the moment the citronella oil was introduced into the chamber and ended at the time when no ant had moved through the exit for 1min. In each experiment, the time when the first ant had completely moved through the exit was counted as time zero. Experimental sessions were repeated six times for each exit size, and image analysis was performed using the slow-motion mode of video-playing software. After each trial, the chamber was washed with water and left to air dry for 3–4 hours until there was no smell of citronella oil. Two methods were adopted to ensure that the chamber was free of citronella: (1) we utilized several chambers of the same size; (2) each chamber used would be used again one week later when the experiment was finished.

In experiment 2 (the control experiment), pure water was added instead of citronella oil. Each control experiment began when the pure water was introduced into the chamber and ended if no ant had escaped for 1min.

## Results

### Evacuation Experiments

**Fig 2:**
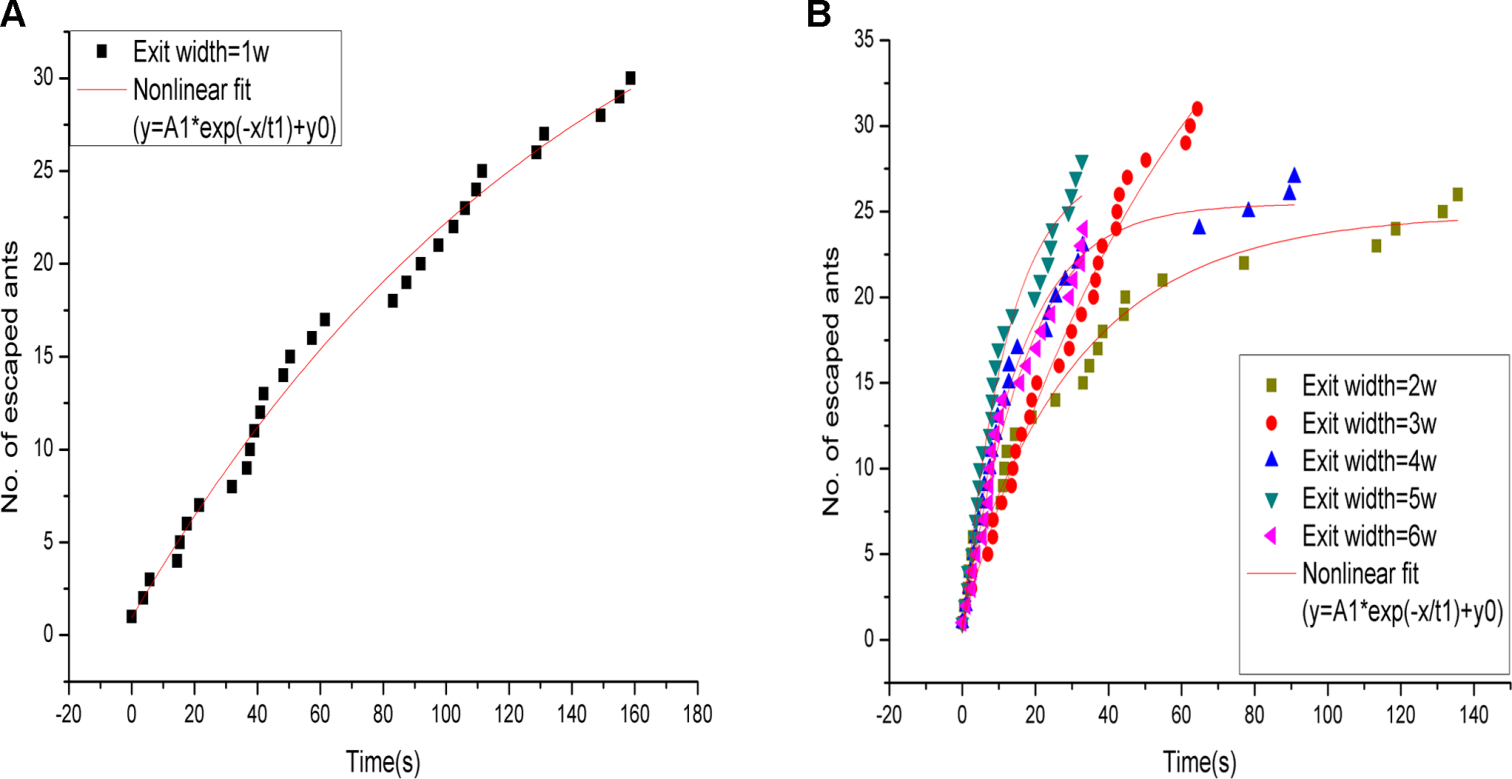
Escape distribution of ants under the effect of a repellent. (A), (B) The temporal evolution of the number of ants escaping through an exit size of 1*w* = 0.5 cm and other exit sizes (2*w* = 1.0 cm, 3*w* = 1.5 cm, 4*w* = 2.0 cm, 5*w* = 2.5 cm, and 6*w* = 3.0 cm); the data of one experimental repetition is shown. The solid line represents the result of nonlinear fitting. The flow of ants is divided into several groups. Solid line: $y=(A1)*\text{exp}(\frac{-x}{t1})+y0$; where y represents the number of escaped ants, and x represents time.

On the basis of the size measurements of 50 randomly selected ants, the average head width was 0.3 ± 0.08 cm (mean ± standard deviation) and body length 1.1 ± 0.16 cm. After taking into consideration the space that the ants’ limbs and antennae would occupy, the exit width of 1*w* was set at 0.5 cm to ensure that the opening allowed only one ant to escape at a time. The temporal evolution of the number of ants passing through the exit was extracted from video records. Fig 2 depicts the results of experiments using various exit widths. Based on nonlinear fitting, the curve shapes of the temporal evolution of the number of ants that escaped through each exit width all showed exponential decay. In addition, the data showed that the ant flow through the exit divided into several groups, indicating that a long time interval existed among the ant groups during the escape period. This result was superficially similar to the group formations of exiting pedestrians [14], but the reasons leading to such group formations were quite different.

In a study by Helbing et al. [14], pedestrians were found to form groups at exits because of jamming; however, our video observations showed that ant groups formed due to long time intervals in the flow of individuals. Soria et al. [11] and Boari et al. [12] also did not observe ant jamming or clogging near exits.

**Fig 3:**
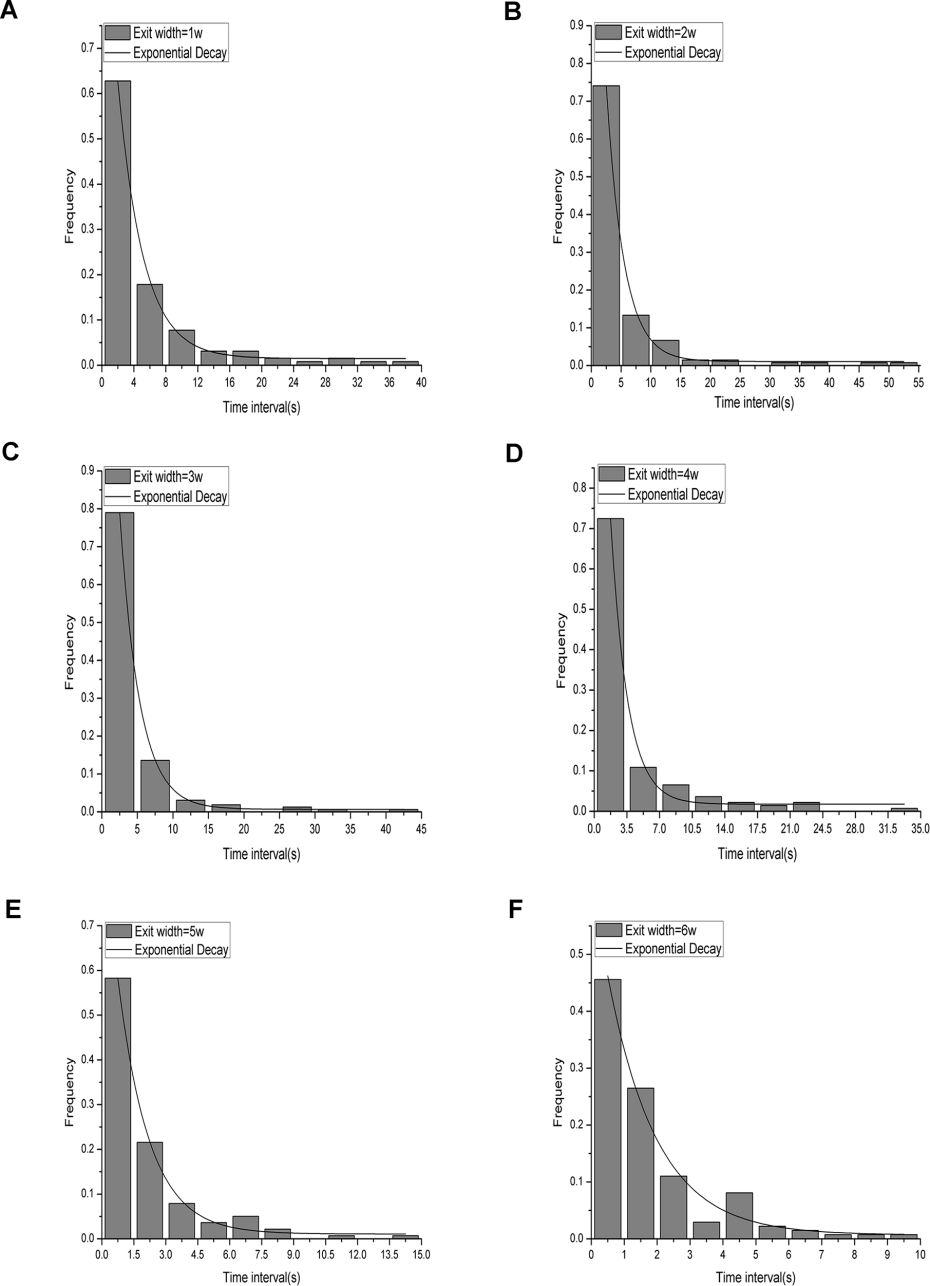
Time interval frequency distribution of all repetitions of the experiments for each exit width. The histogram represents the experimental results for each exit width, and the solid line represents the result of nonlinear fitting. (A), (B), (C), (D), (E), and (F) depict the frequency distributions of the time intervals in the six experimental repetitions for exit sizes of 1*w* = 0.5 cm, 2*w* = 1.0 cm, 3*w* = 1.5 cm, 4*w* = 2.0 cm, 5*w* = 2.5 cm, and 6*w* = 3.0 cm. All of the results show exponential decay based on nonlinear fitting.

To quantitatively study the ant groups, time intervals were measured; the time interval was defined as the time period between one ant completely moving through the exit and the next ant completely moving through the exit and was measured from the first ant until the last one. Fig 3 shows histograms based on the statistics of the time intervals for each exit width for all of the repetitions. The binning value was determined according to the book *Applied statistical methods* [17]. Nonlinear fitting of the data showed that the curve shapes of the results for the different exit widths all demonstrated exponential decay. This differs from findings on humans [18] and granular materials [19], in which the distribution of time intervals followed a power-law rule. Our results can be expressed by the following:

$$p_{f}=\alpha \cdot {\text{e}}^{\frac{-dt}{\beta }}+\varepsilon$$ (Equation 1)

**Fig 4:**
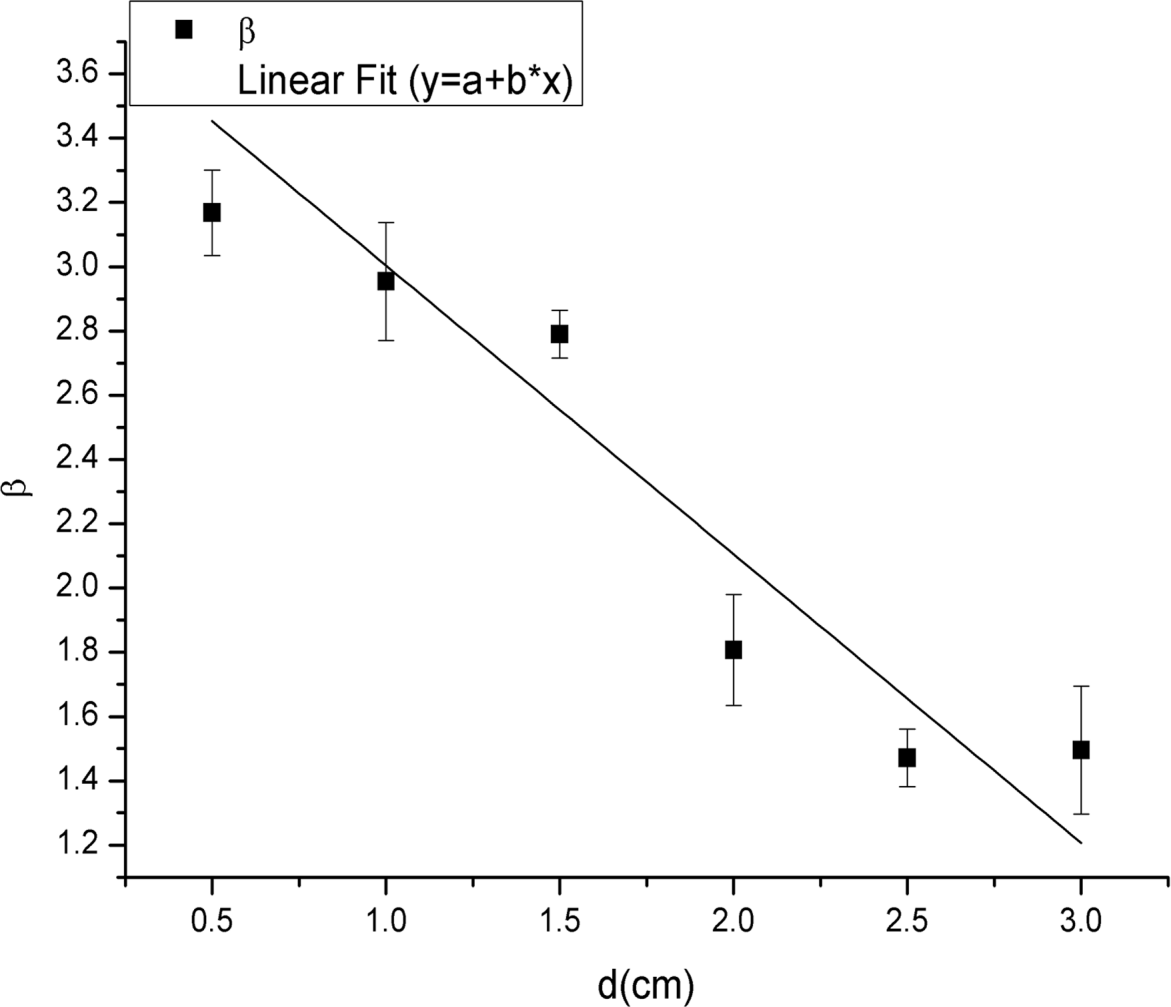
Relationship between the value of *β* and the six different exit widths. The six exit widths were 1*w* (0.5 cm), 2*w* (1.0 cm), 3*w* (1.5 cm), 4*w* (2.0 cm), 5*w* (2.5 cm), and 6*w* (3.0 cm). The square represents the value of *β* for each exit width, and the solid line represents the result of linear fitting. A trend in which the value of *β* decreased as the exit width increased is evident.

**Fig 5:**
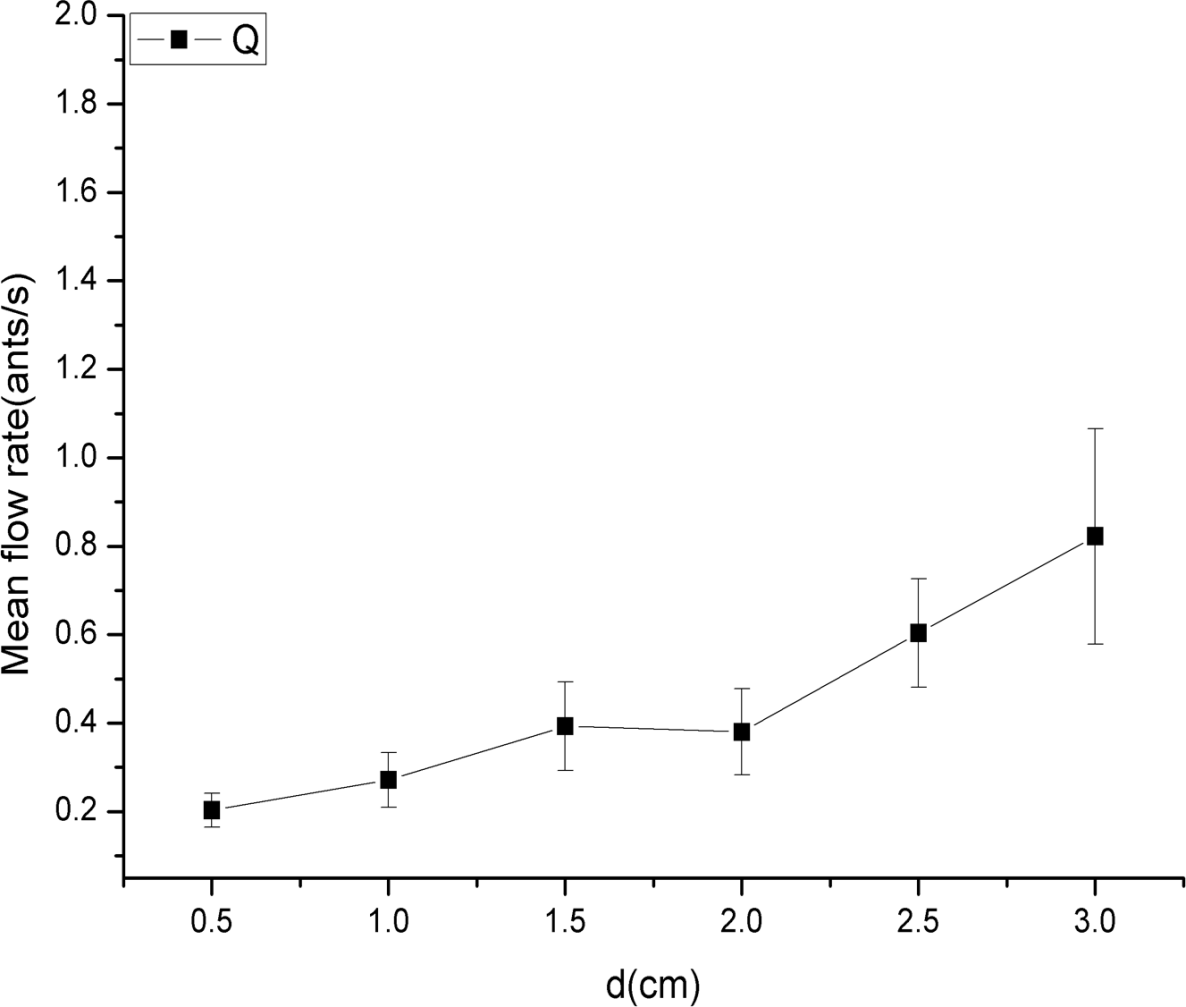
*Q* (mean flow rate) of all of experimental repetitions for the six different exit widths. The six exit widths were 1*w* (0.5 cm), 2*w* (1.0 cm), 3*w* (1.5 cm), 4*w* (2.0 cm), 5*w* (2.5 cm), and 6*w* (3.0 cm). The square represents the *Q* (mean flow rate) for each exit width. The data indicate no obvious linear relationship between *Q* (mean flow rate) and *d* (exit size).

In this equation, *p*_*f*_ denotes the frequency of ants exiting at different time intervals. The nonlinear-fitting parameters (*α*, *β* and *ε*) are constants for each value of *d* (exit width). The parameter *β* is an important quantity for characterizing these distributions. The value of *β* was 3.17, 2.95, 2.79, 1.81, 1.47 and 1.50 for the exit sizes of 1*w* = 0.5 cm, 2*w* = 1.0 cm, 3*w* = 1.5 cm, 4*w* = 2.0 cm, 5*w* = 2.5 cm, and 6*w* = 3.0 cm, respectively. A plot of *β* versus *d* is displayed in Fig 4. A trend in which the value of *β* decreased when *d* increased was evident. As the mean time interval is the inverse of *Q* (the mean flow rate), we explored the relationship between *Q* and *d* (Fig 5) and found no obvious linear relationship between these variables. This result differs from the result found for human evacuation under normal conditions, where the flow rate was found to depend linearly on *d* [20].

The following mathematical expectation values were obtained from these histograms for all of the repetitions for each exit width in Fig 3: 5.66 s, 5.54 s, 4.51 s, 4.21 s, 2.11 s, and 1.77 s for exit widths of 1*w* (0.5 cm), 2*w* (1.0 cm), 3*w* (1.5 cm), 4*w* (2.0 cm), 5*w* (2.5 cm), and 6*w* (3.0 cm), respectively.

**Fig 6:**
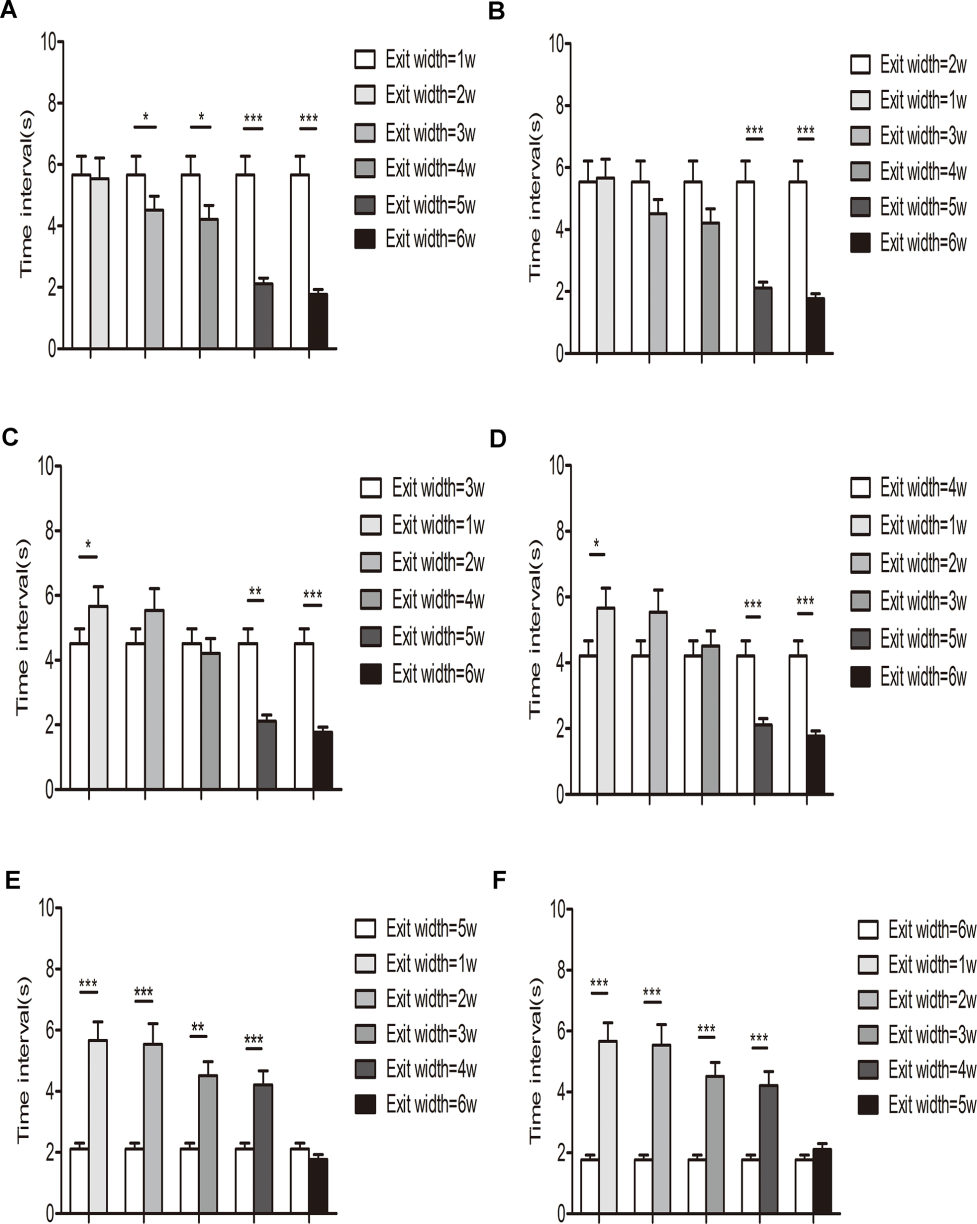
Comparison of time intervals among ant experiments with six exit widths using the t-test. One, two, and three stars indicate a *P*-value below 0.05, 0.01, and 0.001, respectively. (A), (B), (C), (D), (E), and (F) show the results of comparisons between the exit widths 1*w* (0.5 cm), 2*w* (1.0 cm), 3*w* (1.5 cm), 4*w* (2.0 cm), 5*w* (2.5 cm), and 6*w* (3.0 cm), with each exit width being compared with every other exit width.

To further describe the relationship between time interval and different exit width, t-tests comparing all of the time intervals for the six repetitions of two exit widths were performed. The results are shown in Fig 6; one, two, and three stars indicate P-values below 0.05, 0.01, and 0.001, respectively. The three cases indicate a significant difference in the time intervals between the two exit widths over six repetitions, whereas no star indicates no significant difference in the time intervals between exit widths.

The differences in time intervals were greater between exit widths of greater size variability, as demonstrated in Fig 6. For example, in Fig 6A, the result for the 1*w* (0.5 cm) exit width is compared with those for the other five widths, with the mean time interval for the 1*w* being greater than that for the other five widths. In particular, the time intervals for the 3–6*w* exit widths were significantly different from those of 1*w*.

Overall, the time intervals for each exit width differed from one another, and furthermore, the mean time interval decreased with increasing exit width according to the mathematical expectation of the time intervals. The mean time interval expresses the mean flow rate of consecutive ants; thus, the results also show that exit size significantly affected the mean flow rate.

To further study the relationship between ant groupings and flow rate through the exit, we studied the relationship between the number of ants in a group (defined as *S*) and the group flow rate through the exit (defined as *Q*_*S*_). We regarded ants as a group if the time interval between the escape of any two consecutive ants was below the mathematical expectation value for that exit width. We chose the mathematical expectation as the criterion to separate groups because these results would better agree with the phenomena directly represented in the figures. *Q*_*S*_ refers to the group flow rate. For example, for a group of *N* ants,

$$\left\{\begin{array}{l} t_{S}=t_{N}-t_{1}\\ S=N\\ Q_{S}=\frac{N-1}{t_{S}}\\ N\geq 2\\ d\in \{0.5,1.0,1.5,2.0,2.5,3.0\} \end{array}\right\}$$ (Equation 2)

*t*_*S*_ represents the evacuation time of this group, and *t*_1_ and *t*_*N*_ represent the time when the first and *N*th ant in this group completely moved through the exit. It must be noted that we only considered groups containing more than one ant.

**Fig 7:**
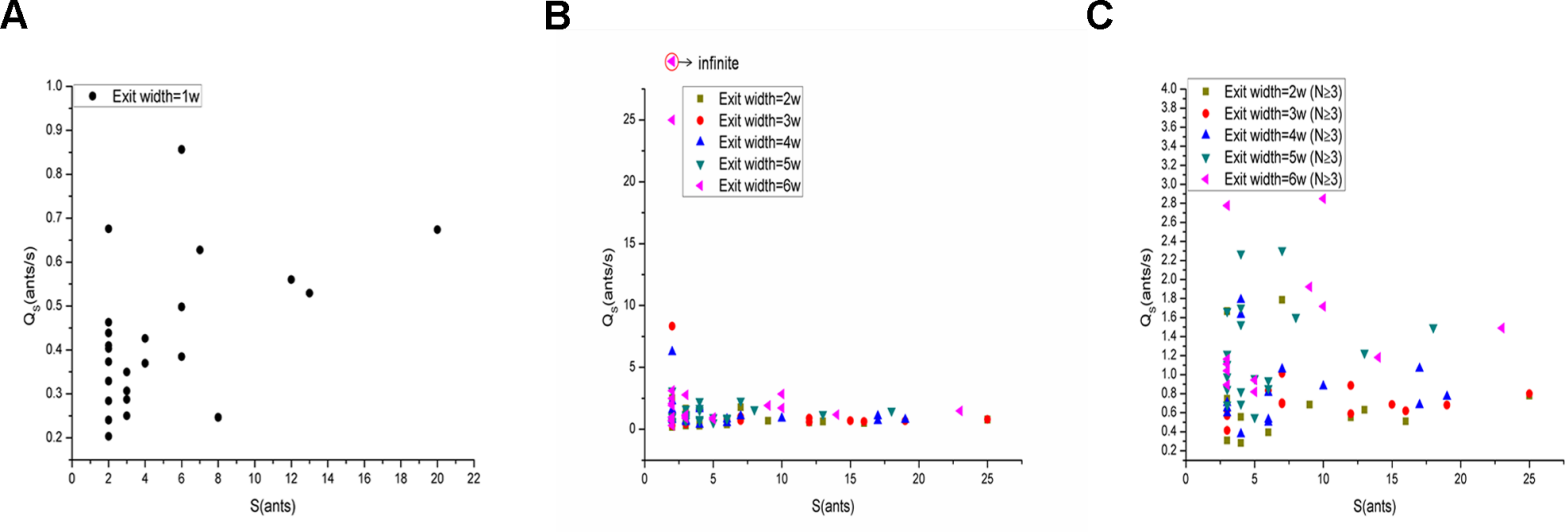
Relationship between the number of ants in a group (*S*) and *Q*_*S*_ through an exit. (A), (B) and (C) show the relationship between *S* and *Q*_***S***_ for the 1*w* (when *N*≥2), 2*w*-6*w* (when *N*≥2) and 2*w*-6*w* (when *N*≥3) exit widths, respectively. (A), (B) and (C) reveal a trend toward an increase in *Q*_***S***_ with increasing *S* only when the exit width was 1*w* (0.5 cm).

Fig 7(A) shows the relationship between *S* and *Q*_*S*_ for the 1*w* exit width. Surprisingly, a trend of increasing *Q*_*S*_ with increasing *S* was evident from the shape of the resulting curve for the 1*w* exit width. The relationship between *S* and *Q*_*S*_ for the 1*w* exit width was studied further by analyzing the correlation between *S* and *Q*_*S*_ in the evacuation experiments using SPSS statistics software (Table 1). The Pearson correlation between *S* and *Q*_*S*_ was 0.489 for the exit width of 1*w* (0.5 cm). The sig. (2-tailed) also indicated that *Q*_*S*_ and *S* were significantly correlated. This result indicates that for the 1*w* exit width, the size of the group affected the group flow rate through the single exit.

**Table 1:** Analysis of the correlation between *Q*_*S*_ and *S* for the 1*w* exit width in the evacuation experiments.

	Pearson Correlation	Sig. (2-tailed)
**Exit width = 1w**	0.489*	0.015

It is shown that *Q*_*S*_ and *S* are significantly correlative.

^a^* Correlation is significant at the 0.05 level (2-tailed).

^b^** Correlation is significant at the 0.01 level (2-tailed).

However, the data presented in Fig 7(B) revealed a singularity for the 2*w*-6*w* exit width when *S* = 2. In fact, we did observe that when a group contained two ants, the two ants escaped at the same time in the experiments. Therefore, t_*N*_-t_1_ = 0 (*N* = 2), and the denominator of this equation would be zero. Thus, *Q*_S_ was infinite. To avoid this singularity, only groups containing more than two ants (that is *N*≥3) were taken into consideration. Fig 7(C) shows the relationship between *S* and *Q*_*S*_ for the 2*w*-6*w* exit width when *N*≥3. There was no obvious trend between *S* and *Q*_*S*_ for the 2*w*-6*w* exit width, as there was for the 1*w* exit width. This might be because the larger exit (exit width = 2*w*-6*w*) allowed multiple ants to egress through the exit at almost the same time.

**Fig 8:**
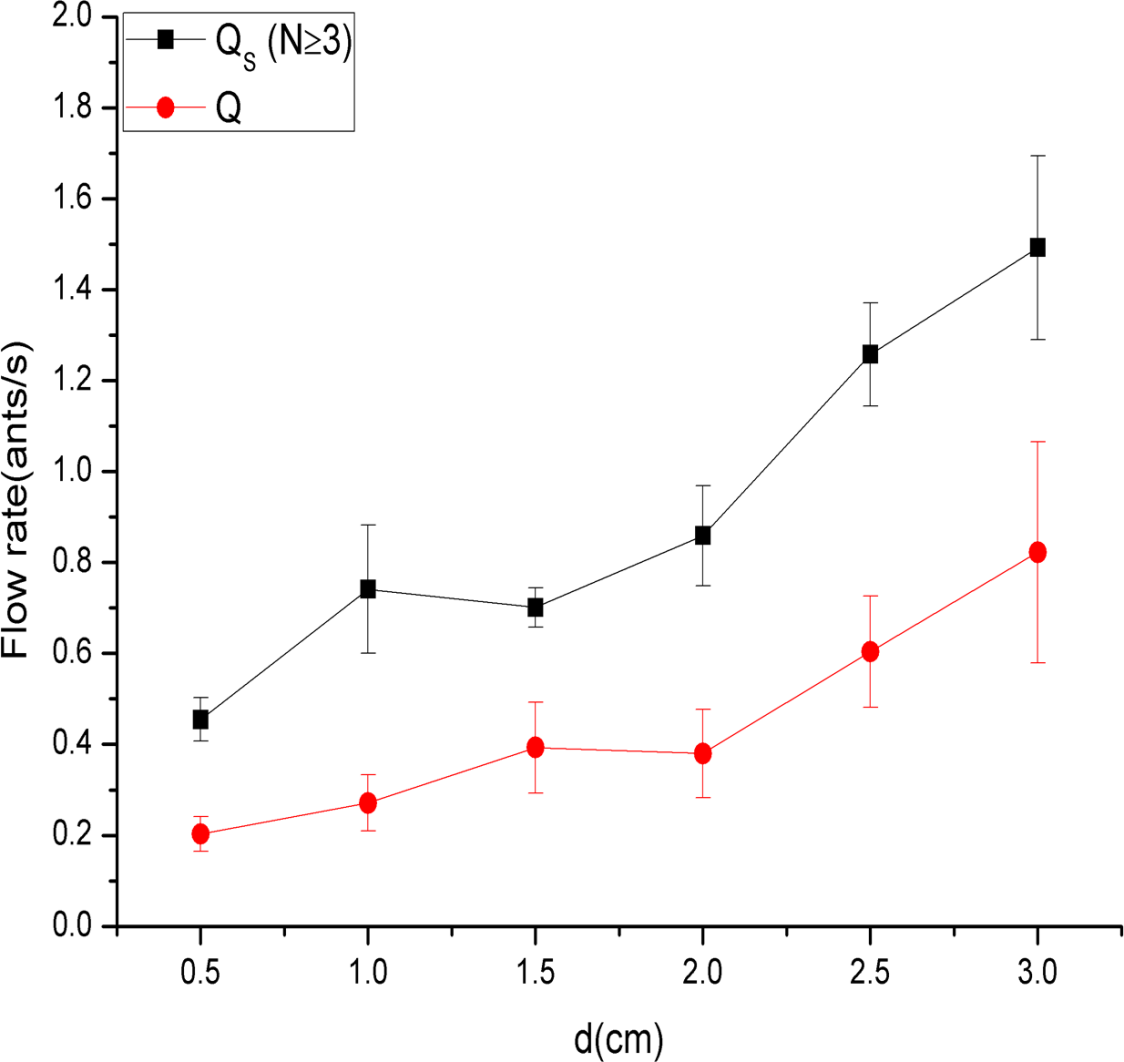
Mean *Q*_*S*_ (*N*≥3) and *Q* for all experimental repetitions for the six different exit widths. The six exit widths were 1*w* (0.5 cm), 2*w* (1.0 cm), 3*w* (1.5 cm), 4*w* (2.0 cm), 5*w* (2.5 cm), and 6*w* (3.0 cm). The square and circle represent the mean *Q*_***S***_ and *Q* for each exit width, respectively. Similar trends exist between mean *Q*_***S***_ and *d* and between *Q* and *d*.

Furthermore, the relationship between mean *Q*_*S*_ (*N*≥3) and *d* is also investigated in Fig 8. A similar trend existed between mean *Q*_*S*_ (group flow rate) (*N*≥3) and *d* and also between *Q* (mean flow rate) and *d*. Indeed, the trend was so obvious that in general, mean *Q*_*S*_ (*N*≥3) (and also Q) increased with increasing *d*. However, with the same value of *d*, the mean flow rate (*Q*) was clearly smaller than the mean group flow rate (*Q*_*S*_) (*N*≥3).

### Control Experiment

**Fig 9:**
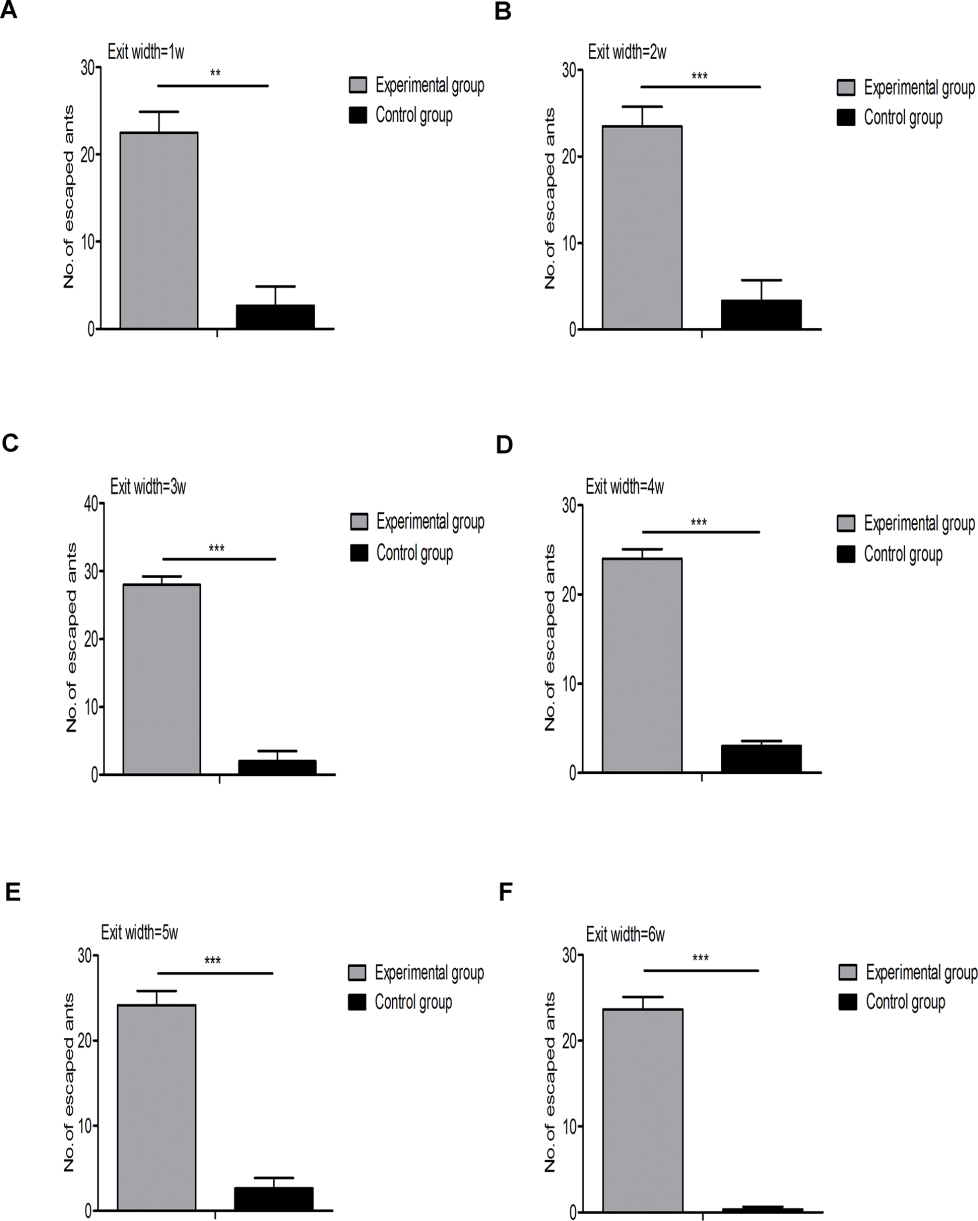
Comparison of the number of escaped ants between treatment and control experiments using the t-test. One, two, and three stars indicate a *P*-value below 0.05, 0.01, and 0.001, respectively. (A), (B), (C), (D), (E), and (F) compare the results of the 1*w* (0.5 cm), 2*w* (1.0 cm), 3*w* (1.5 cm), 4*w* (2.0 cm), 5*w* (2.5 cm), and 6*w* (3.0 cm) exit widths, respectively. Many more ants escaped during the treatment experiments than during the control experiments.

Experiment 2 (the control experiment) was repeated three times and employed a set-up that was identical to that of experiment 1, except that pure water was added instead of citronella oil. To explore the repellent effect of the citronella oil, we extracted from the video records the number of ants that escaped in both experiment 1 and experiment 2 for each exit width. These results, evaluated by t-tests, are presented in Fig 9. The *P*-values were 1.2×10^−3^, 9.1×10^−4^, 4.7×10^−6^, 4.2×10^−6^, 6.6×10^−5^ and 1.2×10^−5^ for the exit widths of 1*w* (0.5 cm), 2*w* (1.0 cm), 3*w* (1.5 cm), 4*w* (2.0 cm), 5*w* (2.5 cm), and 6*w* (3.0 cm), respectively. Many more ants escaped in experiment 1 than in experiment 2, demonstrating the repellent effect of citronella oil on the ants.

The original data underlying the findings of our study are presented in S1 Table.

## Discussion

Previously, the question of how pedestrians escape from a crowd has been simulated by models such as the Social Force model [14], which identified some interesting aspects of pedestrian behavior, such as the “Faster is Slower” effect. This phenomenon was further studied by Parisi and Dorso [21], who showed that the effect resulted from jamming that occurred at an exit. The first experimental evidence of the “Faster is Slower” effect in granular flows was presented by Gago et al. [19]. Subsequently, Garcimartin et al. [18] clearly demonstrated the “Faster is slower” effect in controlled experiments in which students had to evacuate from a room.

A type of behavior, termed “selfish evacuation behavior” [11,12], may be explained by the fact that in very extreme situations, people walk straight toward an exit in an attempt to save their own lives as individuals. This behavior has been found in stress situations in both humans and some animals, such as mice. Saloma et al. [22] studied the egress of mice through an exit of variable width. In their experiment, the mice were placed in a pool of water and had to swim through an exit door to reach a dry platform, and their escape behavior conformed to the numerically predicted [23] exponential and power law frequency distributions of the exit burst size. To overcome some of the limitations of that model, a later agent-based model added the element of copying to provide a more accurate description of the exit behavior of mice from a flooded two-exit chamber. Overall, the results of the two-exit chamber experiment with mice were more consistent with the expectations of numerical simulations than those of the previous model [24].

However, it has been demonstrated that ants do not show “selfish evacuation behavior” with either citronella oil [11] or high temperature [12]. Ants are social insects, and as such, the priority for survival is the colony and not the individual, which differs from the priority of other animals and humans. In this work, the ants stressed with citronella oil exhibited neither “selfish evacuation behavior” nor jamming at the exit. Through further research of ant behavior rules and the group formation phenomenon, we showed that there were many obvious differences between ant and human behaviors. Considering these results, ant egress itself is worth studying due to the efficient evacuation. It appears that the work in this paper cannot be directly related to the egress of humans.

In future work, we will improve the image processing techniques to obtain detailed trajectories and velocities to achieve a better understanding of the behavior of escaping ants in these experiments.

## Conclusions

We investigated the dynamics of ants escaping from a stress condition in a rectangular, single-exit chamber. Our experiments were carried out to study the rules of ant behavior and the phenomenon of group formation. We observed that ant flow separated into several groups, but the reason for this differed from the jamming behavior observed in pedestrian dynamics. To quantitatively study ant grouping, all time intervals were counted, and the frequency distributions of the intervals were found to follow the exponential decay law rather than the power-law observed for human movements. In addition, the mean flow rate did not depend linearly on the exit width, also contrary to the case of humans. Exit size did have an effect on the time intervals, and the number of ants in a group affected the group flow rate for the 1*w* exit width.

In summary, we performed critical experiments to examine the rules of the behavior of ants under stress conditions and the formation of groups. Our experiments provide important insight into the differences between human and ant behaviors. In addition, our work has relevance for studies of animal welfare and ant egress.

## Supporting Information

S1 Table This is the original data underlying the findings of our study. (XLSX)
